# GWAS Follow-up Study Discovers a Novel Genetic Signal on 10q21.2 for Atopic Dermatitis in Chinese Han Population

**DOI:** 10.3389/fgene.2019.00174

**Published:** 2019-03-12

**Authors:** Xin-Ying Cai, Lu Cheng, Chong-Xian Yu, Yan-Yan Wu, Ling Fang, Xiao-Dong Zheng, Fu-Sheng Zhou, Yu-Jun Sheng, Jun Zhu, Jie Zheng, Yuan-Yuan Wu, Feng-Li Xiao

**Affiliations:** ^1^Institute of Dermatology and Department of Dermatology of First Affiliated Hospital, Anhui Medical University, Hefei, China; ^2^Key Laboratory of Dermatology, Anhui Medical University, Ministry of Education, Hefei, China; ^3^State Key Laboratory Incubation Base of Dermatology, Anhui Medical University, Hefei, China

**Keywords:** atopic dermatitis, single-nucleotide polymorphism, susceptibility loci, genome-wide association study, follow-up study

## Abstract

Atopic dermatitis (AD) is a common inflammatory skin disease with high heritability. Two susceptibility loci have been confirmed in our previous AD genome-wide association study (GWAS). To look for additional genetic factors in Chinese Han ethnicity, we performed a large-scale GWAS follow-up study. Forty-nine top single nucleotide polymorphisms (SNPs) that had never been reported previously were genotyped using Sequenom Massarray system in an independent cohort, which consist of northern Chinese (1634 cases and 1263 controls) and southern Chinese (2985 cases and 9526 controls). Association analyses were performed using PLINK 2 software. Three SNPs in northern and ten SNPs in southern were found exhibiting association evidence with AD (*P* < 0.05). Finally, SNP rs224108 on 10q21.2 showed high significance for AD in joint analysis of GWAS and replication study (*P*_*meta*_ = 4.55 × 10^−9^, OR = 1.21), and was confirmed as an independent genetic marker by Linkage disequilibrium calculation and conditional logistic regression analysis. Bioinformatics analysis strongly suggested that rs224108 may have the potential to alter the target gene expression through non-coding epigenetic regulation effects. Meanwhile, SNP rs11150780 on 17q25.3 was also found suggestive association with AD (*P*_*meta*_ = 7.64 × 10^−7^, OR = 1.18). Our findings confirmed a novel susceptibility signal on 10q21.2 for AD in Chinese Han population and advanced the understanding of the genetic contribution to AD.

## Introduction

Atopic dermatitis (AD) is a chronic inflammatory skin disease and characterized by intense itching and recurrent eczematous lesions. It's a global health problem affecting up to 20% of children and 3% of adults worldwide (Nutten, 2015). In China, the prevalence of clinical diagnosed AD in children aged 1–7 years is 12.94% (Guo et al., 2016). The pathogenesis of AD is not entirely clear, which involves a complex interaction of genetic and environmental factors (Kim, 2015; Kantor and Silverberg, 2017; Thorsteinsdottir et al., 2019).

During recent years, a series of studies have discovered a number of genetic susceptibility factors related to AD. Notably, several recent genome-wide association studies (GWASs) in multiple ethnic groups have identified 38 susceptibility loci (Esparza-Gordillo et al., 2009, 2013; Paternoster et al., 2011, 2015; Sun et al., 2011; Hirota et al., 2012; Ellinghaus et al., 2013; Weidinger et al., 2013; Kim et al., 2015; Marenholz et al., 2015; Schaarschmidt et al., 2015), providing vital clues for understanding the underlying genetic basis of AD. Most of these loci were shown to be associated with skin epithelial function and innate/adaptive immune response, highlighting the two major pathogenetic pathways of AD. However, these genetic signals account for only a minor part of risk effects, suggesting that additional genetic factors remain to be discovered.

**Figure 1:**
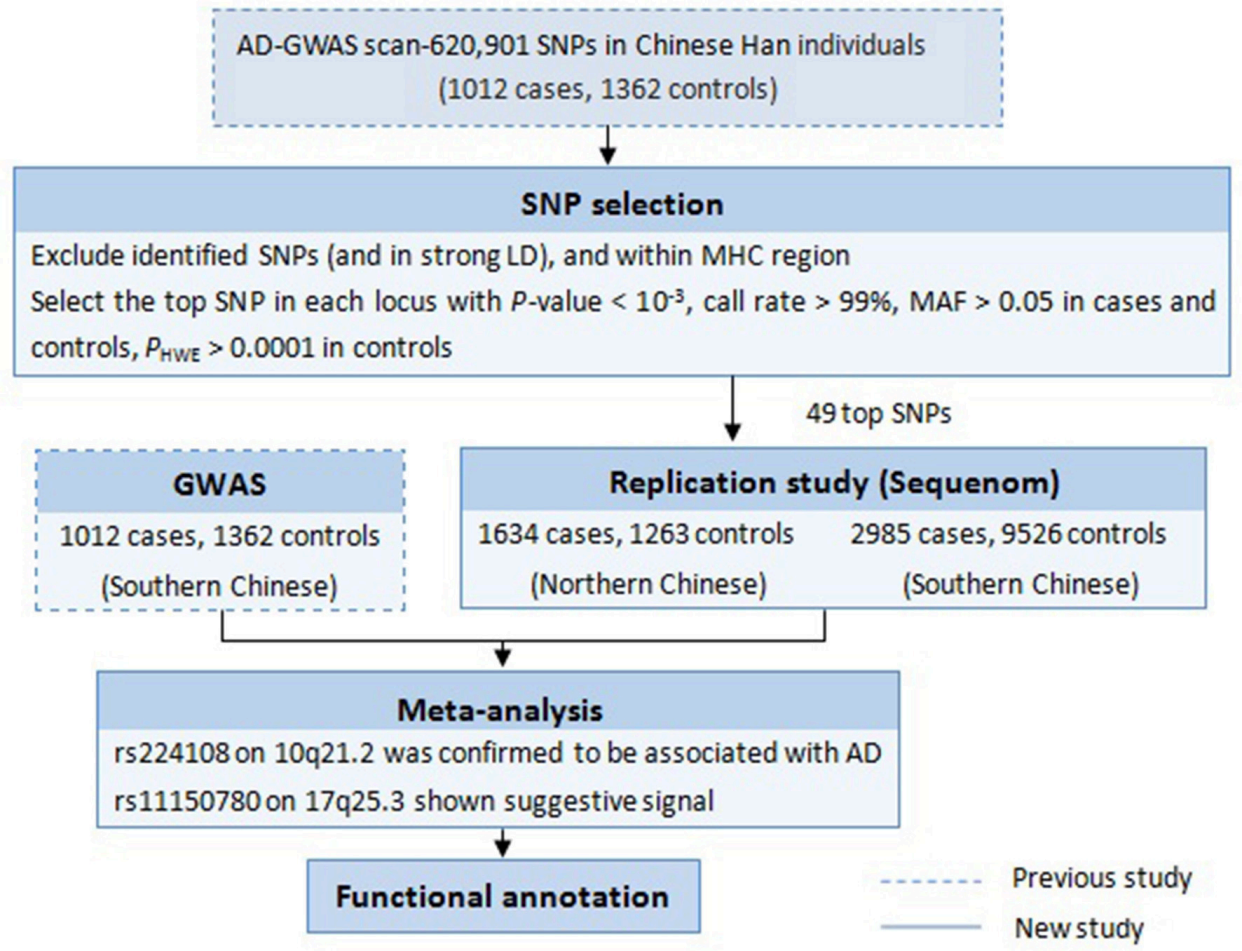
Workflow of the study design.

Follow-up study is one of the most effective GWAS data mining methods, which focuses on the previously overlooked susceptibility variants. Such strategies used in our lab have successfully discovered additional new susceptibility loci for some diseases, like vitiligo and systemic lupus erythematosus (Sheng et al., 2011; Tang et al., 2013; Zhu et al., 2015). In 2011, our group conducted the first AD-GWAS in Chinese Han population, and identified two new susceptibility loci (5q22.1 and 20q13.33) and a suggestive locus (10q21.2) (Sun et al., 2011). In the next year, the Japanese GWAS added eight novel susceptibility loci including 10q21.2 (Hirota et al., 2012). On this basis, to look for additional Chinese specific disease loci for AD, we performed a follow-up study in an independent larger cohort, taking advantage of our initial published AD-GWAS datasets. The experimental design is summarized in Figure 1.

## Materials and Methods

### Subjects

The discovery stage of the study consisted of 1012 AD cases and 1362 healthy controls of Chinese Han ancestry from our previously published GWAS (Sun et al., 2011). The independent replication samples were recruited from multiple regions in China, which consist of northern Chinese (replication 1: 1634cases and 1263controls) and southern Chinese (replication 2: 2985cases and 9526controls) (Table 1; Supplementary Table 1). All the cases with AD were diagnosed by at least two experienced dermatologists using the Hanifin & Rajka criteria. All the controls were healthy individuals without AD, other atopic diseases, systemic disorders or family history of atopic diseases (including first-, second- and third-degree relatives). Clinical information was collected from both cases and controls through a structured questionnaire. Informed consent was obtained from all participants or their legal guardians. EDTA anticoagulated venous blood samples were collected from all individuals. The study was approved by the Institutional Ethical Committee of Anhui Medical University and was conducted according to the Declaration of Helsinki principles.

**Table 1:** Summary of samples used in GWAS and replication study.

**Stage**	**Cases**			**Controls**		
	**Sample size**	**Mean age (s.d.)**	**Male/female(%)**	**Sample size**	**Mean age (s.d.)**	**Male/female(%)**
GWAS^a^	1012	5.01 (7.63)	58.1/41.9	1362	33.10 (13.60)	55.4/44.6
Replication 1^b^	1634	5.97 (8.50)	58.6/41.4	1263	30.31 (12.82)	46.8/53.2
Replication 2^c^	2985	5.57 (7.25)	62.0/38.0	9526	27.58 (12.74)	54.4/45.6
Total	5631	5.59 (7.71)	60.4/39.6	12151	28.49 (12.97)	53.7/46.3

a *GWAS samples are from Southern China*.

b *Replication 1 samples are from Northern China*.

c *Replication 2 samples are from Southern China*.

Genomic DNA was extracted from peripheral blood mononuclear cells by standard procedures using Flexi Gene DNA kits (Qiagen, Germany) and was diluted to working concentrations of 50 ng/μl for genome-wide genotyping and 15-20 ng/μl for the replication study.

### SNP Selection for Replication

First, we focused on the 759 SNPs that reach a *P-*value < 10^−3^ in the discovery phase of our previous AD-GWAS (Sun et al., 2011). Second, SNPs were selected with very clear genotyping clusters, call rates > 99%, minor allele frequency (MAF) > 0.05 in both cases and controls, *P*-value for Hardy-Weinberg equilibrium (HWE) > 0.0001 in controls. After excluding the SNPs in strong linkage disequilibrium (LD) (*r*^2^ ≤ 0.5) with any previously confirmed AD risk variants and within the major histocompatibility complex region, we choosed the most significant SNP in each locus (If there were multiple SNPs in strong LD with a strong association in one region, the SNP with the smallest *P*-value was selected). Finally, a tolal of 49 top SNPs were selected for replication study.

### Genotyping

The genome-wide genotyping in the discovery stage was conducted using Illumina Human610-Quad BeadChips (Illumina, Inc., San Diego, CA, USA) (Sun et al., 2011) and genotyping in the replication stage was performed using Sequenom Massarray system (Sequenom, Inc., San Diego, CA, USA) at the Key Laboratory of Dermatology at Anhui Medical University. In replication stage, locus-specific PCR and detection primers were designed using the MassARRAY Assay Design 3.0 software (Sequenom). The DNA samples were amplified by multiplex PCR reactions, and the PCR products were then used for locus-specific single-base extension reactions. The resulting products were desalted and transferred to a 384-element SpectroCHIP array. Allele detection was performed using MALDI-TOF MS. The mass spectrograms were analyzed by the MassARRAY Typer software (Sequenom).

### Statistical Analysis

Quality control was performed in each dataset separately using PLINK 2 software. The concordance rate between the genotypes from the Illumina and the Sequenom analyses was > 99%. SNPs was required with a call rate > 90% in cases or controls and *P*_HWE_ > 0.0001 in controls. Cluster patterns of the genotyping data from the Illumina and Sequenom analyses were checked to confirm their quality.

Statistical significance was calculated by Chi-square tests, to obtain *P*-values, odds ratios (ORs), and 95% confidence intervals (CIs) for the SNPs using PLINK 2 software. Meta-analysis was conducted using METAL to combine the data from primary GWAS and validation study. Since the study was based on initial GWAS, statistical threshold of joint *P* value for significant associated SNPs was set at 5 × 10^−8^.

In addition, linkage disequilibrium calculation and conditional logistic regression analysis were conducted using PLINK 2 software to determine whether independent effect was existed for the newly identified variant. The information of the SNPs was obtained from 1000 genome phase 3 and previous AD-GWAS data.

### Bioinformatics Analysis

To explore whether the replicated susceptibility loci have potential regulatory functions, we performed a series of bioinformatic analyses. The online tools HaploRegv4.1 (https://pubs.broadinstitute.org/mammals/haploreg/haploreg.php) and Roadmap Epigenomics (http://www.roadmapepigenomics.org/), as well as ENCODE data were used to retrieve regulatory elements of interest, including histone marks, protein-binding site and matched motifs. The rSNPBase (http://rsnp.psych.ac.cn/) database was used to search for regulatory SNPs with experimentally validated regulatory elements controlling transcriptional and post-transcriptional events. Multiple publically available algorithms were employed to predict the functional impact of non-coding genetic variants, such as GWAVA (https://www.sanger.ac.uk/sanger/StatGen_Gwava), FATHMM (http://fathmm.biocompute.org.uk/index.html) and RegulomeDB (http://www.regulomedb.org). The expression and methylation quantitative trait loci (QTL) analyses were performed using publically available data, such as GTEx (http://www.gtexportal.org/home/), eQTLGen (http://www.eqtlgen.org/) and mQTL (http://www.mqtldb.org/) to assess if the SNPs altered gene expression and regulation in relevant normal tissues, such as skin and whole blood.

## Result

### Association Results of GWAS, Replication and Combined Studies

In this replication study, 49 SNPs were genotyped in 4619 AD cases and 10,789 healthy controls. Three SNPs in northern and 10 SNPs in southern were found exhibiting association evidence with AD (*P* < 0.05) (Supplementary Table 2). When the genotypic data from the primary GWAS and the replication stage were combined by meta-analysis, we found that the significance of association at rs224108 exceeded the genome-wide threshold (*P*_meta_ = 4.55 × 10^−9^, OR = 1.21), and another SNP rs11150780 showed suggestive association with AD (*P*_meta_ = 7.64 × 10^−7^, OR = 1.18) (Table 2). However, no evidence of association was observed for the rest SNPs in the meta-analysis. The statistical results of the tested 49 SNPs are summarized in Supplementary Table 2.

**Table 2:** Association evidence for two SNPs in our previous GWAS, replication and combined studies.

				**GWAS**				**Replication 1 (northern)**				**Replication 2 (southern)**				**Meta**	
				**1012 cases and 1362 controls**				**1634 cases and 1263 controls**				**2985 cases and 9526 controls**				**5631 cases and 12,151 controls**	
**SNP**	**Chr**	**Gene**	**Allele**	**MAF**		**P**	**OR (95%CI)**	**MAF**		**P**	**OR (95%CI)**	**MAF**		**P**	**OR (95%CI)**	**P**	**OR (95%CI)**
				**Case**	**Control**			**Case**	**Control**			**Case**	**Control**				
rs224108	10q21.2	ADO/EGR2	C/T	0.1586	0.1211	2.07E-04	1.37 (1.16–1.61)	0.1556	0.1434	1.99E-01	1.10 (0.95–1.28)	0.1387	0.124	3.03E-03	1.14 (1.05–1.24)	4.55E-09	1.21 (1.14–1.29)
rs11150780	17q25.3	SLC38A10	G/A	0.1464	0.1105	2.27E-04	1.38 (1.16–1.64)	0.1301	0.1121	3.86E-02	1.19 (1.01–1.39)	0.1328	0.1183	2.84E-03	1.14 (1.05–1.25)	7.64E-07	1.18 (1.11–1.26)

*Chr, chromosome. MAF, minor allele frequency. Allele, Minor allele/major allele*.

Intriguingly, rs224108 didn't show association with AD in northern samples (*P* = 1.99 × 10^−1^, OR = 1.10), and did actually show effect in southern (*P* = 3.03 × 10^−3^, OR = 1.14). This could be because of the very small sample size of the northern replication cohort. Further studies are needed to confirm the heterogeneity of this association between northern and southern Chinese populations.

### Linkage Disequilibrium and Conditional Logistic Regression Analysis

In order to confirm whether the significance of the newly identified genetic marker (rs224108) was independent or affected by the previously known AD risk SNPs, LD calculation and conditional logistic regression analysis were performed. The SNP rs224108 showed low LDs with rs2393903 (*r*^2^ = 0.10) and rs10995251 (*r*^2^ = 0.005) based on 1000 genome phase 3 data. After controlling for the genetic effect of the reported SNP rs2393903 using conditional logistic regression analysis based on our previous AD-GWAS data, SNP rs224108 was still significantly associated with AD, indicating that the signal of rs224108 was independent (*P* < 0.05, Supplementary Table 3).

### Bioinformatics Analysis for Functional Annotation

**Figure 2:**
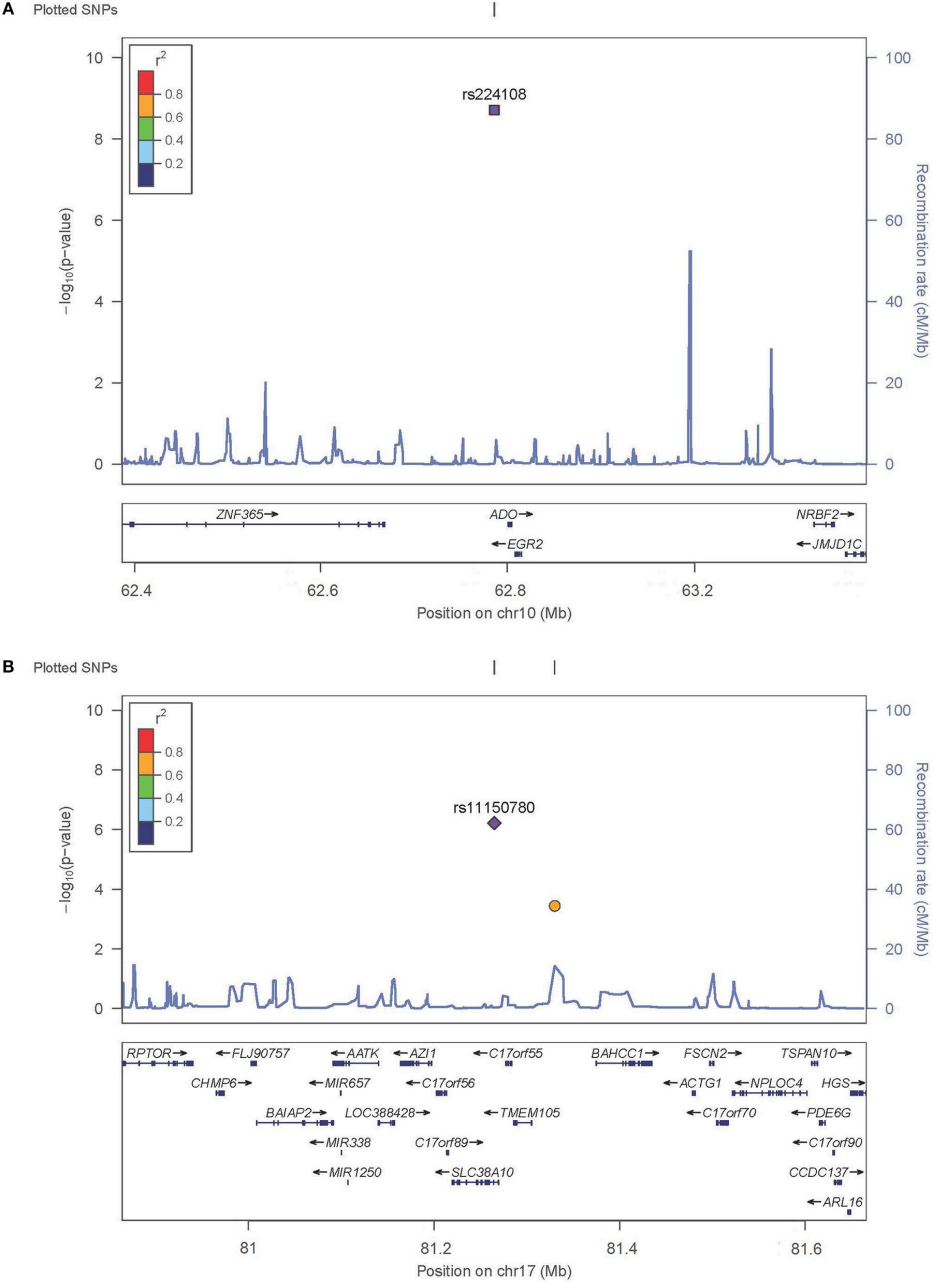
Regional plots for associated SNPs. The *P*-values of SNPs shown as –log_10_ values (y axis) were presented against their chromosomal positions (x axis). Estimated recombination rates were plotted in light blue. SNPs showed strong signal were labeled out. **(A)** Regional plots for rs224108 on 10q21.2. **(B)** Regional plots for rs1150789 on 17q25.3.

SNP rs224108 locates 15 kb upstream of *ADO* and 22 kb downstream of *EGR2* (Figure 2). According to the regulatory annotation information from Haploreg v4, the SNP is within a region with enhancer histone H3K4mel in keratinocyte primary cell. In addition, rs224108 is also within the region of binding site of GATA2 transcription factor based on Chip-Seq (chromatin immunoprecip itation followed by sequencing) data of the ENCODE project. Furthermore, rs224108 was found to overlap a potential consensus TATA binding motif within the binding sites of the GATA2 transcription factor. Collectively, these findings suggest that the genetic region containing rs224108 has the potential to change chromatin architecture via protein binding. Based on the functional annotation information from the rSNPBase database, rs224108 was predicted to have distal regulatory roles. In the eQTL analyses, rs224108 and its highly correlated SNPs (*r*^2^ > 0.8) were associated with the expression level of *ADO* in blood (*P* = 7.39 × 10^−17^) (Võsa et al., 2018). These functional annotation results suggest that the genetic association effect at rs224108 may be due to its impact on the transcriptional regulation of *ADO* (Supplementary Table 4).

SNP rs11150780 is located in an intron of *SLC38A10* (Figure 2). Based on Roadmap Epigenomics data, the SNP appears to map to a regulatory region enriched with enhancer histone marks (H3K4me1, H3K4me3, H3K27ac and H3K9ac) in skin and blood. We also found that the rs11150780 G allele may alter several DNA binding motifs of homeobox transcriptional factors and proteins, including BRCA1, ERalpha-a, and FAC1 (ENCODE and HaploRegv4). Furthermore, the rSNPBase database revealed that rs11150780 involved in proximal and distal transcriptional regulation, and RNA binding protein mediated regulation. According to the QTL analyses, rs11150780 was significantly associated with *SLC38A10* in blood *P* = 4.630 × 10^−59^ (Võsa et al., 2018), and effected on DNA methylation in blood (Gaunt et al., 2016) (Supplementary Tables 5,6).

## Discussion

We conducted a follow-up study to our previous AD-GWAS by performing a validation analysis, genotyping the most promising SNPs in a large independent Chinese cohort including 4,619 AD cases and 10,789 healthy controls. Two variants were validated and showed significant association evidence in meta-analysis of GWAS and replication study: rs224108 (*P*_meta_ = 4.55 × 10^−9^, OR = 1.21) and rs11150780 (*P*_meta_ = 7.64 × 10^−7^, OR = 1.18).

SNP rs224108 is located at the region of 10q21.2. It is 169kb downstream of SNP rs2393903, which was reported to show suggestive association with AD in our previous GWAS (*P* = 1.05 × 10^−7^) (Sun et al., 2011), and 151kb downstream of SNP rs10995251, which was reported in Japanese AD-GWAS (*P* = 5.58 × 10^−20^) (Hirota et al., 2012). However, LD between rs224108 and rs2393903 is very weak (*r*^2^ = 0.10), and LD between rs224108 and rs10995251 is virtually absent (*r*^2^ = 0.005). In addition, to confirm whether the significance of the newly identified genetic signal was independent or influenced by the previously reported SNP rs2393903 in Chinese Han population, conditional analysis were conducted. As a result, SNP rs224108 showed an independent genetic effect on AD.

SNP rs224108 was found to be a functional variant using the public available databases. Based on Haploreg v4, rs224108 appeared to map to a regulatory region with H3K4mel histone. ENCODE annotation showed that rs224108 C risk allele might affect the binding of GATA2 transcription factor by damaging the TATA binding motif. GATA2 transcription factor is a zinc finger DNA-binding protein which potentially inhibits the transcription of downstream genes combining with the promoter region of target genes (Yang et al., 2014). According to the rSNPBase database, rs224108 was predicted to have distal regulatory roles. Moreover, the eQTL study revealed that rs224108 and its highly correlated SNPs might affect *ADO* expression (GTEx Consortium, 2015; Võsa et al., 2018). Taken together, these findings suggest that rs224108 may have the potential to alter the target gene expression through non-coding epigenetic regulation effects.

As for 10q21.2, several genes were observed in the association interval, and the most significantly associated SNP, rs224108, was located at an intergenic region 15 kb upstream of *ADO* and 22 kb downstream of *EGR2*. *ADO* encodes 2-aminoethanethiol dioxygenase which possesses cysteamine dioxygenase activity and involves in the cysteamine metabolism. The cysteamine can reduce airway hypersensitivity, inflammatory cell recruitment and T-helper cell differentiation in allergic asthma (Oh et al., 2013). Additionally, cystamine, a dimeric form of cysteamine, can ameliorate IgE-induced passive cutaneous anaphylaxis and phorbol myristic acid-induced atopic dermatitis (Kim et al., 2010). The other gene, *EGR2*, also seems to be interesting because of its important role in manipulation the peripheral immune response. *EGR2* encodes early growth response 2 protein which is a zinc-finger transcription factor belonging to the Egr family. Egr2 protein is essential for the maintenance of humoral immune tolerance by negatively regulating T cell activation (Morita et al., 2016; Miao et al., 2017). Egr2 inhibits IFN-γ and IL-2 secretion by T cells (Li et al., 2012; Singh et al., 2017), regulates Th17 differentiation and expression of IL-17 (Miao et al., 2013), and binds directly to the promoter of the cell cycle inhibitor p21^cip1^ in T cells (Zhu et al., 2008). *EGR2* has also been associated with susceptibility to Crohn's disease (Rioux et al., 2007), Behcet's disease (Takeuchi et al., 2017), and systemic lupus erythematosus (Myouzen et al., 2010). Combined, all the evidence supported that *ADO* and *EGR2* might likely be the susceptibility genes for AD. However, further fine mapping and further functional studies should be warranted to prove this hypothesis.

SNP rs11150780 also showed suggestive association with AD. It was located at 17q25.3 and in intron 1 of the *SLC38A10* gene. Functional annotation using public databases provided strong evidence for the role of rs11150780 in regulating gene expression. The eQTL analyses showed significant association between rs11150780 and the *SLC38A10* gene in whole blood (GTEx Consortium, 2015; Võsa et al., 2018), and mQTL analyses suggested rs11150780 mighted effect on DNA methylation in blood (Gaunt et al., 2016). *SLC38A10* encodes an amino acid transporter belonging to the functional class of SLC38 family. SLC38A10 protein mediates bidirectional transport of glutamine, glutamate, and aspartate (Hellsten et al., 2017). Further investigation is needed to better understand the contribution of *SLC38A10* in AD pathogenesis.

In summary, in this large-scale GWAS follow-up study, a novel genetic signal on 10q21.2 (rs224108) was confirmed to be strongly associated with AD risk in Chinese Han population. Meanwhile, 17q25.3 (rs11150780) was found to be a suggestive susceptibility locus for AD. Although further studies should be required to gain better insights into the genetic etiology and pathophysiology of AD, our findings have further expanded understanding of the genetic contribution to AD.

## Data Availability

All datasets generated for this study are included in the manuscript and/or the supplementary files.

## Author Contributions

F-LX conceived and designed the study and revised the manuscript. X-YC analyzed the data and drafted the manuscript. LC helped with the interpretation of the results and writing of the manuscript. C-XY, Y-YW, and LF enrolled patients, extracted and diluted DNA and conducted PCR. X-DZ performed bioinformatics' and statistical analysis. F-SZ and Y-JS helped in the SNP selection and genotyping. JunZ, JieZ, and Y-YW participated in the experimental.

### Conflict of Interest Statement

The authors declare that the research was conducted in the absence of any commercial or financial relationships that could be construed as a potential conflict of interest.
