# Supplementary material for: GWAS Follow-up Study Discovers a Novel Genetic Signal on 10q21.2 for Atopic Dermatitis in Chinese Han Population
Source: Front Genet. 2019 Mar 12;10:174. doi: 10.3389/fgene.2019.00174 (PMC6422937; doi:10.3389/fgene.2019.00174)
Supplement: Supplementary file 3 [file Table_3.doc]

| **Supplementary Table 3** The results of conditional regression analysis. | | | | | | |  |  | |  | | |
| --- | --- | --- | --- | --- | --- | --- | --- | --- | --- | --- | --- | --- |
|  |  |  |  |  | | |  |  | |  | | |
| Locus | SNP | r^2^ | Condition on SNP | | | | | | | |  | |
|  |  |  | rs224108 | |  | rs2393903 | | | | |  | |
|  |  |  | *P* | OR(95%CI) |  | *P* | | | OR(95%CI) | |  | |
| 10q21.2 | rs2393903* | 0.10 | 5.20E-04 | 1.24(1.10-1.40) |  |  | | |  | |  | |
|  | rs224108 |  |  |  |  | 1.14E-02 | | | 1.26(1.05-1.50) | |  | |
|  |  |  |  |  | | |  |  | |  | |  |
| *SNP showing suggestive association with AD in our previous GWAS. | | | | | | | | | | | | |
